# Functional microRNA high throughput screening reveals miR-9 as a central regulator of liver oncogenesis by affecting the PPARA-CDH1 pathway

**DOI:** 10.1186/s12885-015-1562-9

**Published:** 2015-07-24

**Authors:** Alexandra Drakaki, Maria Hatziapostolou, Christos Polytarchou, Christina Vorvis, George A. Poultsides, John Souglakos, Vassilis Georgoulias, Dimitrios Iliopoulos

**Affiliations:** 1 Division of Hematology/Oncology, David Geffen School of Medicine, University of California, Los Angeles, Los Angeles, CA USA; 2 Laboratory of Tumor Biology, Department of Medical Oncology, University Hospital of Heraklion, Heraklion, Crete Greece; 3 Center for Systems Biomedicine, Division of Digestive Diseases, David Geffen School of Medicine, University of California, Los Angeles, 650 Charles E. Young Dr., CHS 44-133, Los Angeles, CA 90095-7278 USA; 4 Department of Surgery, Stanford School of Medicine, Stanford University, Palo Alto, CA USA

**Keywords:** miR-9, Hepatocellular oncogenesis, Functional screen, PPARA, E-cadherin

## Abstract

**Background:**

Hepatocellular carcinoma (HCC) is the second leading cause of cancer-related deaths, reflecting the aggressiveness of this type of cancer and the absence of effective therapeutic regimens. MicroRNAs have been involved in the pathogenesis of different types of cancers, including liver cancer. Our aim was to identify microRNAs that have both functional and clinical relevance in HCC and examine their downstream signaling effectors.

**Methods:**

MicroRNA and gene expression levels were measured by quantitative real-time PCR in HCC tumors and controls. A TargetScan algorithm was used to identify miR-9 downstream direct targets.

**Results:**

A high-throughput screen of the human microRNAome revealed 28 microRNAs as regulators of liver cancer cell invasiveness. MiR-9, miR-21 and miR-224 were the top inducers of HCC invasiveness and also their expression was increased in HCC relative to control liver tissues. Integration of the microRNA screen and expression data revealed miR-9 as the top microRNA, having both functional and clinical significance. MiR-9 levels correlated with HCC tumor stage and miR-9 overexpression induced SNU-449 and HepG2 cell growth, invasiveness and their ability to form colonies in soft agar. Bioinformatics and 3′UTR luciferase analyses identified E-cadherin (CDH1) and peroxisome proliferator-activated receptor alpha (PPARA) as direct downstream effectors of miR-9 activity. Inhibition of PPARA suppressed CDH1 mRNA levels, suggesting that miR-9 regulates CDH1 expression directly through binding in its 3′UTR and indirectly through PPARA. On the other hand, miR-9 inhibition of overexpression suppressed HCC tumorigenicity and invasiveness. PPARA and CDH1 mRNA levels were decreased in HCC relative to controls and were inversely correlated with miR-9 levels.

**Conclusions:**

Taken together, this study revealed the involvement of the miR-9/PPARA/CDH1 signaling pathway in HCC oncogenesis.

**Electronic supplementary material:**

The online version of this article (doi:10.1186/s12885-015-1562-9) contains supplementary material, which is available to authorized users.

## Background

Hepatocellular cancer (HCC) is the most frequent type of malignancy originating from the liver with a recently rising incidence in the United States [1]. It is the second most common cause of cancer-related death worldwide with more than 500,000 new cases per year. The incidence of the disease approximates the death rate, which reflects the aggressiveness of this tumor [2]. HCC is one of the few types of cancer in which the various risk factors are well characterized. Specifically, infections with the hepatitis B and C virus as well as aflatoxin B1 (AFB) are responsible for almost 80 % of the cases [3].

At the same time, the molecular mechanisms that lead to the pathogenesis of HCC are not completely understood. Up to date, there are several genes involved in the signaling pathways essential for the initiation and progression of hepatocellular carcinogenesis and these include, but are not limited to, c-myc, PTEN, e-cadherin, cyclin D1 and p53 [4].

MicroRNAs are small non-coding RNA molecules, 18–25 nt long, that act as negative regulators of gene expression, through binding in the 3′UTR of the coding sequence of genes [5]. Previous studies have identified different microRNAs to be deregulated in liver pre-cancerous and cancer stages [6, 7]. Specifically, microRNAs have been identified to regulate cell cycle through regulation of cyclin G1 [8]. In addition, miR-21 was identified to have a potent oncogenic potential in HCC by blocking directly the PTEN tumor suppressor gene [9]. Furthermore, another study revealed a 20-microRNA metastasis signature that could significantly predict primary HCC tissues with venous metastases from metastasis-free solitary tumors with 10-fold cross-validation [10]. Interestingly, Xu Y et al. showed that a polymorphism in the promoter region of miR-34b/c was associated with an increased risk for primary hepatocellular carcinoma [11]. Also, serum microRNAs were found to potentially serve as biomarkers for HBV infection and diagnosis of HBV-positive HCC [12], suggesting the potential of measuring circulating microRNA levels as biomarkers in HCC. However, it has not been extensively studied which microRNAs have both clinical and functional relevance in this type of cancer. Here, we are describing that miR-9 is potentially a novel oncogene in liver cancer, regulating the tumor initiation, growth and metastatic potential of liver cancer cells. On the other hand, inhibition of miR-9 expression blocks the tumor properties of liver cancer cells, including cell growth and migration, suggesting its therapeutic potential. Interestingly, we found that miR-9 suppressed CDH1 mRNA expression levels, directly through binding in its 3′UTR and indirectly through regulation of PPARA expression levels. Taken together, this study reveals a novel role for the miR-9/PPARA/CDH1 signaling pathway in HCC oncogenesis.

## Methods

### RNA from HCC and liver control samples

RNA was extracted from 24 Fixed-Formalin- Paraffin-Embedded (FFPE) HCC and 14 liver control (adjacent non-tumor) tissue specimens obtained from consenting patients in the Department of Surgery at Stanford University and were approved by the Ethics Committee of the Stanford University Medical School.

### MicroRNA library screen

SNU-449 liver cancer cells were plated in 96-well plates and transfected with a microRNA library consisting of 316 microRNA mimics and 2 negative control microRNAs (100 nM) (Dharmacon Inc). At 48 h post-transfection, SNU-449 cell invasiveness was evaluated in Boyden chamber invasion plates. Assays were conducted according to manufacturer’s protocol, using 2 % FBS as a chemoattractant. Invading cells were fixed and stained with 0.1 % crystal violet, 24 h post seeding. The cells that migrated through the filter were quantified by counting the entire area of each filter. MicroRNAs that affected >2-fold (50 %) SNU-449 invasiveness relative to microRNA negative control treated SNU-449 cells were considered as positive hits.

### Invasion assay

We performed invasion assays in SNU-449 cells 24 h after transfection with miR-9 or anti-miR-9 and their respective controls. Invasion in matrigel has been conducted by using standardized conditions with BD BioCoat Matrigel invasion chambers (BD Biosciences). Assays were conducted according to manufacturer’s protocol, using 2 % FBS as the chemoattractant. Non-invading cells on the top side of the membrane were removed, while invading cells were fixed and stained with 0.1 % crystal violet, 24 h post-seeding. The cells that migrated through the filter were quantified by counting the entire area of each filter, using a grid and an Optech microscope at a 20× magnification.

### Real-time PCR analysis

Quantitative real-time RT-PCR was performed to determine the expression levels of miR-9, miR-21 and miR-224 in 24 human HCC (stage I *n* = 5; stage II *n* = 9; stage III *n* = 6; stage IV *n* = 4) and 11 liver control tissues. RNA was isolated using Trizol, according to manufacturer’s instructions (Invitrogen). Real-time RT-PCR was assessed on a CFX384 detection system (BioRad) using the Exiqon PCR primer sets according to manufacturer’s instructions. MicroRNA expression levels were normalized to the levels of U6 small nuclear snRNA (203907, Exiqon). Normalized miRNA levels were quantified relative to the levels of a given control tissue. Real-time PCR was employed to determine the expression levels of CDH1, PPARA, vimentin and PDK4. Reverse transcription was carried out using the Retroscript Kit (AM1710, Applied Biosystems). Real-time PCR was carried out using the IQ SYBR Green Supermix (170–8882, BioRad). Gene expression levels were normalized to the levels of Glyceraldehyde-3-phosphate dehydrogenase (GAPDH) and β-actin. Normalized gene expression levels were quantified to the respective control. The sequences of the primers used are the following:

CDH1-F: 5′-TGAAGGTGACAGAGCCTCTGGAT-3′

CDH1-R: 5′-TGGGTGAATTCGGGCTTGTT-3′

PPARA-F: 5′-GGCGAGGATAGTTCTGGAAGC-3′

PPARA-R: 5′-CACAGGATAAGTCACCGAGGAG -3′

Vimentin-F: 5′-CCAAACTTTTCCTCCCTGAACC -3′

Vimentin-R: 5′-GTGATGCTGAGAAGTTTCGTTGA -3′

PDK4-F: 5′-CCCCGAGAGGTGGAGCAT-3′

PDK4-R: 5′-GCATTTTCTGAACCAAAGTCCAGTA-3′

### Colony formation assay

SNU-449 and HepG2 liver cancer cell lines were transfected with miR-9 or anti-miR-9 and their respective controls. Then, triplicate samples of 2×10^5^ cells from each cell line were assayed for colony formation using the CytoSelect Cell Transformation kit (Cell Biolabs, Inc). The number of colonies were counted after 7 days.

### Cell growth assay

SNU-449 and HepG2 liver cancer cell lines were transfected with miR-9 or the respective control and plated on a 96-well plate (5×10^3^ cells/well). 48 and 72 h later, cell growth was assessed using the Cell-Titer Glo Luminescence Cell Viability Assay (Promega).

### Liver tumor sphere formation assay

SNU-449 liver cancer cell lines were transfected with miR-9 or anti-miR-9 were plated in ultra-low attachment plates (Corning), 24 h post-transfection and were grown in DMEM F12 (Invitrogen) medium supplemented with B-27 (Gibco), bFGF and EGF in the culture medium containing 1 % methyl cellulose to prevent cell aggregation. The number of spheres was evaluated 6 days post plating.

### 3′UTR luciferase assay

SNU-449 cells were transfected with the reporter vectors carrying the 3′UTR of CDH1 (cat. no 25038, Addgene) or PPARA (cat. no HmiT054001-MT06, Genecopoeia). The constructs harbored the seed sequence of miR-9 (wildtype) or had a deletion of this sequence (miR-9 mutant). At 24 h, they were transfected with miR-9 or miR-control and at 48 h luciferase activity was measured using the Dual Luciferase Reporter Assay System (Promega).

### Statistical analysis

All experiments were performed in triplicate unless otherwise stated. Statistical analyses were performed with the use of Origin software, version 8.6. Student’s *t*-test was used to examine the statistical difference in miR-9 expression between control and HCC tissues. The correlation significance was determined by means of Spearman and Pearson correlation analyses. A *P*-value of 0.05 or less was considered statistically significant.

## Results

### Strategy for the identification of functional and clinically relevant microRNAs in hepatocellular (HCC) oncogenesis

**Fig. 1:**
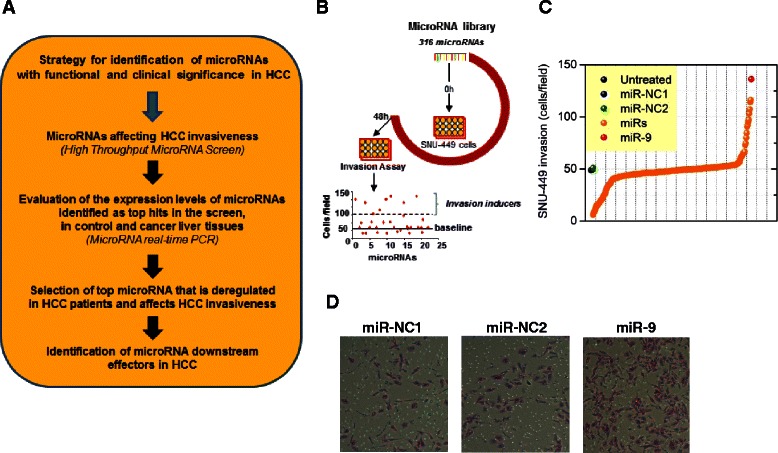
High-throughput screening identifies microRNAs that control HCC invasiveness. **a** Steps followed for identification of microRNAs with both functional and clinical significance in HCC. **b** Strategy workflow: A library of 316 microRNAs was transfected in SNU-449 liver cancer cells and their invasiveness was measured 48 h post transfection in Boyden chamber invasion plates. **c** Screen data plotted as different microRNAs transfected in SNU-449 cells (x-axis) and their invasiveness (cells/field) compared to scrambled sequence controls (no effect, value = 50) (y-axis). The red circle represents miR-9, while the blue and yellow circles the microRNA negative controls (miR-NC1, miR-NC2). **d** SNU-449 cells stained with crystal violet in BioCoat Matrigel invasion chambers after treatment with miR-NC1, miR-NC and miR-9. Invading cells were fixed and stained with 0.1 % crystal violet, 24 h post-seeding. The cells that migrated through the filter were quantified by counting the entire area of each filter, using a grid and an Optech microscope at a 20X magnification

The identification of microRNAs that are differentially expressed between HCC and control tissues cannot predict which of these microRNAs are functionally important in HCC pathogenesis, while on the other hand the identification of microRNAs affecting liver cancer cellular properties does not always suggest that these microRNAs would have human relevance. Thus, we have developed an experimental strategy, aiming to reveal the microRNAs that have both functional and human relevance in HCC (Fig. 1a). Specifically, we followed a dual experimental approach by performing first a high-throughput microRNA screen in SNU-449 liver cancer cells and secondly, evaluated the expression levels of the microRNAs derived from this screen in human liver cancer and control tissues.

### Identification of microRNAs regulating HCC invasiveness by performing a human microRNAome library screen in liver cancer cells

We were interested in identifying the top microRNAs that were functioning as activators or suppressors of HCC invasiveness. To address this question, we performed a microRNA library screen in SNU-449 liver cancer cells. Specifically, we transfected a library of 316 microRNAs and two microRNA negative controls (miR-NC) and 48 h post transfection, SNU-449 cell invasiveness was measured by performing a cell invasion Boyden chamber assay (Fig. 1b). MicroRNAs that induced >2-fold SNU-449 invasiveness were characterized as microRNA invasion inducers and the microRNAs that suppressed >2-fold SNU-449 invasiveness were named as microRNA invasion suppressors (Fig. 1c). Our screen revealed five microRNAs (miR-9, -224, -21, -24, -27a) as HCC invasion inducers and 23 microRNAs (miR-29a, -145, -29b, -507, -26a, -122a, -375, -195, -203, -26b, -199b, -125a, -223, -1, -101, -199a, -124a, -125b, let-7b, let-7a, miR-148a, -152, -148b) as HCC invasion suppressors. Overexpression of miR-9 was found to be the top inducer of SNU-449 cell invasiveness (Fig. 1d).

### Expression levels of microRNAs, acting as invasion inducers, in HCC patient tissues

**Fig. 2:**
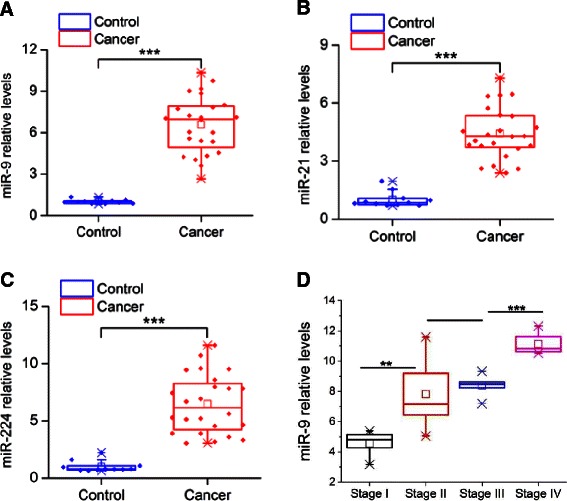
Relative microRNA expression levels in HCC and liver control tissues. **a** MiR-9, (**b**) miR-21 and (**c**) miR-224 expression levels in 24 HCC tumors and 11 control liver tissues assessed by real-time RT-PCR analysis. **d** MiR-9 expression levels in different stages of HCC tumors relative to controls. Data are represented as mean ± SE. ***P < 0.001, in comparison to control

Due to the fact that we were interested in studying a microRNA that we could therapeutically target by a microRNA inhibitor, we focused our interest on the microRNAs that acted as inducers of HCC invasiveness. The screen above revealed that the top three microRNAs as statistically significant inducers of liver cancer cell invasiveness were miR-9, miR-224 and miR-21. Thus, we evaluated their expression levels in 24 HCC tumors and 11 liver control tissues by real-time quantitative PCR analysis. MiR-9 was found to be 6.5-fold up-regulated in HCC relative to control tissues (Fig. 2a) and miR-21 expression levels were increased 4.4-fold in HCC relative to controls (Fig. 2b). In addition, miR-224 was found to be 6.4-fold up-regulated in HCC relative to controls (Fig. 2c). Next, we have examined if there is any correlation between miR-9, miR-21 and miR-224 expression levels and HCC tumor stage. MiR-9 levels were found to increase during HCC progression (Fig. 2d), having lower levels in early stages (stage I) and increasing until late stages (IV). MiR-21 expression was statistically different between stage I and II HCC tumors, while miR-224 expression was not statistically different between different HCC tumor stages (Additional file 1: Figure S1).

The next step was to integrate the microRNA library and tissue profiling data. This analysis revealed that miR-9 is the microRNA that has the highest ability to induce HCC invasiveness, it is highly expressed in HCC tumors and its expression correlates with HCC tumor stage, suggesting both its functional and human relevance in HCC.

### MiR-9 is an inducer of HCC cancer cell properties

**Fig. 3:**
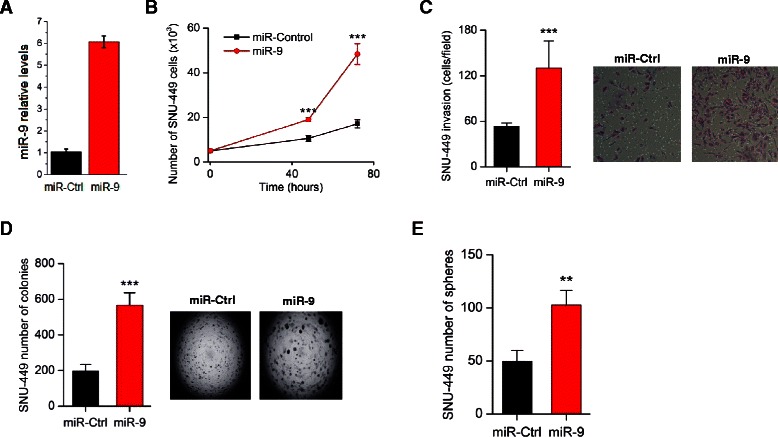
Effects of miR-9 overexpression on liver cancer cellular properties. **a** Relative miR-9 expression levels in SNU-449 cells after transfection with miR control or miR-9, 48 h post-transfection. **b** Cell growth of SNU-449 liver cancer cells transfected with miR negative control (miR-Control) or miR-9, 48 h and 72 h post-transfection. **c** Invasion of SNU-449 after transfection with miR negative control (miR-Control) or miR-9, 48 h post-transfection. **d** Soft agar colony assay in SNU-449 overexpressing miR-9 or miR-Control. **e** Effects of miR-9 overexpression on the number of SNU-449 liver tumor spheres. All data are represented as mean ± SE. ***P < 0.001, **P < 0.01

To evaluate the oncogenic potential of miR-9 activity in HCC, we performed a series of cancer cell assays, by overexpressing miR-9 in SNU-449 and HepG2 liver cancer cell lines (Fig. 3a, Additional file 1: Figure S2a). First, we examined if miR-9 affects liver cancer cell growth properties. Specifically, miR-9 was overexpressed in SNU-449 and HepG2 liver cancer cells and the total cell number was measured 48 and 72 h post-transfection (Fig. 3b, Additional file 1: Figure S2b). We found that miR-9 induced liver cancer cell growth in both cell lines, more significantly 72 h post transfection. Second, we studied miR-9 effects on HCC invasiveness. MiR-9 overexpression induced SNU-449 invasiveness (Fig. 3c, Additional file 1: Figure S2c), consistent with our primary microRNA library screen analysis. Furthermore, miR-9 overexpression induced ~2.3-fold HepG2 cell invasiveness, revealing that the effects of miR-9 on liver cancer cell invasiveness are not SNU-449 cell line specific. Third, miR-9 overexpression induced significantly the ability of both SNU-449 and HepG2 cells to form colonies in soft agar (Fig. 3d, Additional file 1: Figure S2d). Finally, due to the fact that miR-9 may function as an oncogene, we examined its ability to regulate liver tumor sphere formation. We found that miR-9 overexpression increased the ability of SNU-449 cells to form spheres in suspension (Fig. 3e). Taken together, these functional assays suggest that miR-9 plays an oncogenic role in HCC, affecting both cancer cell proliferation and invasiveness rates.

### PPARA and E-cadherin (CDH1) as direct downstream targets of miR-9 in HCC

**Fig. 4:**
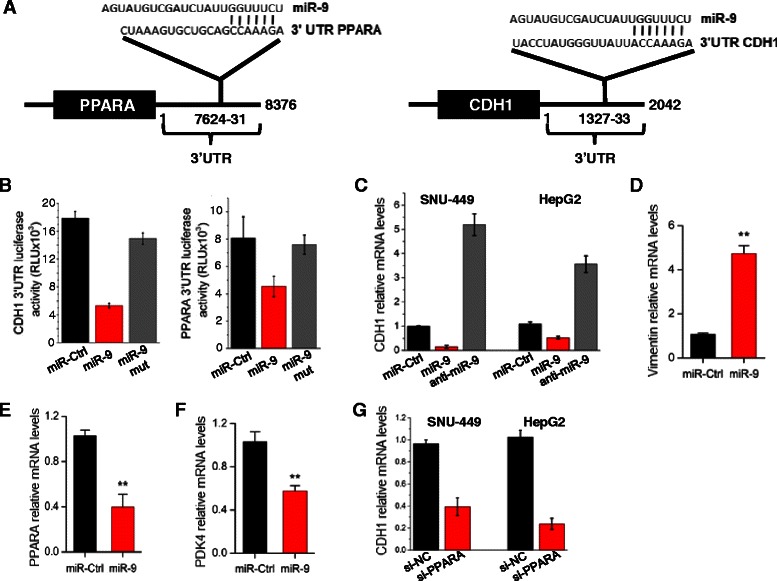
CDH1 and PPARA as direct targets of miR-9 in HCC. **a** Sequence complementarity between miR-9 seed sequence and the 3′UTRs of PPARA and CDH1. **b** CDH1 and PPARA 3′UTR luciferase assay activity in SNU-449 cells transfected with miR-Ctrl or miR-9, 48 h post-transfection. MiR-9 sequence was wildtype or mutated (miR-9 mut). **c** CDH1 mRNA levels in SNU-449 and HepG2 cells transfected with miR-9 or anti-miR-9, 48 h post-transfection, assessed by real-time RT-PCR. **d** Vimentin, (**e**) PPARA and (**f**) PDK4 mRNA levels in SNU-449 cells transfected with miR-9, 48 h post-transfection, assessed by real-time PCR. **g** CDH1 mRNA levels in SNU-449 and HepG2 cells transfected with an siRNA against PPARA (siPPARA) or an siRNA negative control (siCtrl), 48 h post-transfection. All data are represented as mean ± SE. ***P < 0.001, **P < 0.01, *P < 0.05

We were interested in examining the downstream gene effectors of miR-9 oncogenic activity in HCC. Bioinformatics analysis by using the TargetScan algorithm revealed that miR-9 has very strong and highly conserved binding sites on the 3′ untranslated regions (UTRs) of PPARA and CDH1 genes. Specifically, miR-9 has sequence complementarity in the position 7624-31 nt of the 3′UTR of PPARA and also in the position 1327-33 nt of the 3′UTR of CDH1 (Fig. 4a). To examine the direct interactions between miR-9 and these potential downstream direct targets, we performed 3′UTR luciferase assays. MiR-9 was overexpressed in SNU-449 cells that were co-transfected with a construct harboring the 3′UTR of PPARA or CDH1 under luciferase activity. We found that miR-9 overexpression suppressed both CDH1 and PPARA 3′UTR luciferase activities, having a stronger effect on CDH1 (Fig. 4b). Mutation of the miR-9 binding sites in the 3′UTR PPARA and CDH1 luciferase vectors abolished the suppressive effects of miR-9. These data validate at the molecular level of the direct interactions between miR-9 and PPARA or CDH1 genes.

Next, we examined miR-9 effects on CDH1 mRNA expression levels. Overexpression of miR-9 suppressed significantly CDH1 mRNA levels, while inhibition of miR-9 expression by an antisense-miR-9 resulted in up-regulated of CDH1 mRNA levels, in both SNU-449 and HepG2 liver cancer cells, assessed by qPCR analysis (Fig. 4c). Due to the fact that CDH1 is an epithelial marker gene [13] and its loss has been correlated with epithelial mesenchymal transition, we examined the expression levels of the mesenchymal marker [14], vimentin. Real-time PCR analysis showed that miR-9 overexpression increased significantly vimentin mRNA levels (Fig. 4d). In addition, miR-9 overexpression reduced PPARA mRNA levels in SNU-449 cells (Fig. 4e). To further validate the miR-9/PPARA interaction, we examined PDK4 expression levels after miR-9 overexpression in liver cancer cells. PDK4 is a known downstream direct target of the PPARA transcription factor in hepatocytes [15, 16]. MiR-9 overexpression resulted in ~50 % reduction of PDK4 mRNA levels, assessed by real-time PCR analysis (Fig. 4f). Previous studies have identified a positive correlation between E-cadherin and the PPARA signaling pathways [17, 18]. So, we inhibited PPARA expression levels using an siRNA against PPARA (siPPARA) in SNU-449 and HepG2 cells and assessed levels of CDH1 mRNA by real-time PCR. Inhibition of PPARA resulted in >60 % reduction in CDH1 mRNA expression levels in both cell lines (Fig. 4g). Taken together, these data suggest that miR-9 regulates CDH1 expression directly through binding to its 3′UTR and indirectly by controlling PPARA expression. PPARA inhibition resulted in suppression of CDH1 mRNA levels, while CDH1 inhibition, by using an siRNA against CDH1, did not affect PPARA mRNA levels (Additional file 1: Figure S3), suggesting that there is not a bi-directional regulation between PPARA and CDH1.

### Suppression of the miR-9 signaling pathway on HCC cell properties

**Fig. 5:**
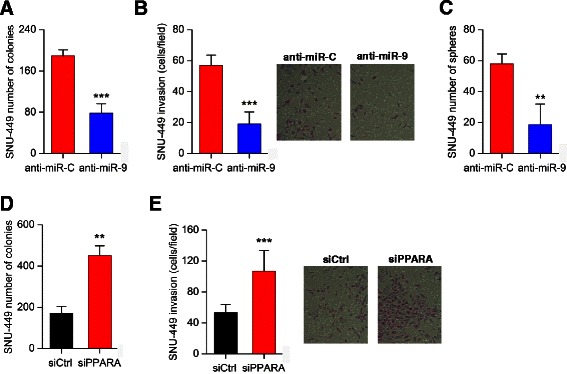
Effects of miR-9 inhibition on liver cancer cellular properties. **a** Soft-agar colony formation assay; (**b**) cellular invasion assay and (**c**) tumor sphere formation assay in SNU-449 cells transfected with an antisense microRNA negative control (anti-miR-C) or an antisense microRNA-9 (anti-miR-9). **d** Effects of PPARA inhibition by an siRNA (siPPARA) or an siRNA negative control (siCtrl) on the ability of SNU-449 cells to form colonies in soft-agar and (**e**) SNU-449 cell invasiveness. All data are represented as mean ± SE. ***P < 0.001, **P < 0.01

To evaluate the therapeutic potential of miR-9 in HCC oncogenesis, we used an anti-sense miR-9 molecule (anti-miR-9) and performed a series of experiments. First, we found that miR-9 inhibition suppressed significantly the ability of SNU-449 cells to form colonies in soft agar (Fig. 5a), reduced their invasiveness (Fig. 5b) and also their ability to form liver tumor spheres (Fig. 5c). All these data reveal the therapeutic potential of targeting miR-9 in liver cancer. To further evaluate these findings, we examined the effects of PPARA inhibition on liver cancer cells. We found that inhibition of PPARA expression, by an siRNA (siPPARA), induced the ability of SNU-449 cells to form colonies in soft agar (Fig. 5d) and increased their cellular invasiveness (Fig. 5e), suggesting that PPARA has a tumor suppressive function in HCC.

### MiR-9/PPARA/CDH1 pathway expression levels in HCC tissues

**Fig. 6:**
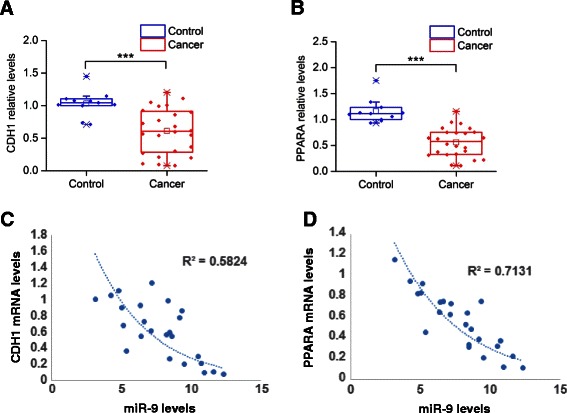
MiR-9 signaling pathway levels in HCC tissues. **a** CDH1 and (**b**) PPARA mRNA relative expression levels in 24 HCC tumors and 11 control liver tissues assessed by real-time RT-PCR analysis. Gene expression levels were normalized to the levels of GAPDH and β-actin. Normalized gene expression levels were quantified relative to the levels of a given control tissue. **c** Correlation analysis between miR-9 and CDH1 mRNA levels in 24 HCC tissues. **d** Correlation analysis between miR-9 and PPARA mRNA levels in 24 HCC tissues. Data are represented as mean ± SE. ***P < 0.001, in comparison to control

To study the human relevance of the miR-9/PPARA/CDH1 signaling pathway, we examined PPARA and CDH1 expression levels in 24 HCC and 11 control liver tissues. Real-time PCR analysis showed that CDH1 had >40 % down-regulation of its mRNA levels in HCC relative to controls (Fig. 6a) and PPARA had >50 % reduced levels in HCC relative to control tissues (Fig. 6b). Furthermore, we performed correlation analysis, evaluating the significance of correlation between miR-9 and PPARA or CDH1 mRNA levels in HCC tissues. Consistent with our *in vitro* findings, miR-9 was inversely correlated with both CDH1 (R^2^ = 0.5824) (Fig. 6c) and PPARA (R^2^ = 0.7131) (Fig. 6d) mRNA levels in HCC tissues. Taken together, these findings reveal the human relevance of the miR-9 signaling pathway in HCC oncogenesis.

## Discussion

Different signaling pathways have been implicated in HCC pathogenesis [19], however the role of non-coding RNAs has not been studied extensively until recently. Non-coding RNAs consist primarily of the microRNAs and long non-coding RNA (lincRNAs) and several studies have implicated their role in HCC initiation and progression [6, 7, 20, 21]. Specific microRNA signatures have been identified to be deregulated in HCC patient tissues and also to correlate with different clinicopathological parameters [10, 22]. Furthermore, microRNAs have been associated with hepatitis infection, cirrhosis and patient survival [23].

In this study, we have screened the human microRNAome, aiming to identify microRNAs that are potent regulators of HCC invasiveness. Interestingly, we found 28 microRNAs to affect significantly (>2-fold) the invasiveness of SNU-449 liver cancer cells. Five of these microRNAs behaved as HCC invasion inducers, while 23 microRNAs as HCC invasion suppressors. This screen revealed novel microRNAs potentially involved in HCC pathogenesis and also validated findings from previous studies. Specifically, microRNAs such as miR-21, miR-29a/b, miR-26a, miR-101, miR-122a, miR-124a, miR-375 and let-7a/b have been correlated with HCC pathogenesis through regulation of essential signaling pathways [9, 24–30]. More recently, we have identified that miR-24 is part of a feedback loop circuit involved in HCC pathogenesis [7]. On the other hand the role of miR-9, miR-148b, miR-203 and miR-507 in HCC pathobiology is not well understood. Recently, high miR-9 expression levels were found to be correlated with poor prognosis in HCC patients [31]. Furthermore, miR-148b expression was found to be decreased in HCC patients [32], however it is not known which signaling pathways are mediators of miR-148b activity in HCC. In addition, it has been shown that miR-203 is suppressed in HCC tissues due to DNA methylation on its regulatory area [33]. Finally, nothing is known regarding the role of miR-507 in HCC pathogenesis.

Here, we provide evidence that miR-9 affects different liver cancer cell properties, including liver tumor sphere formation. When liver cancer cells are placed in low attachment plates or in suspension, they have the ability to form liver tumor spheres, which potentially represent the cellular population harboring tumor-initiating properties [34, 35]. Here, we evaluated for the first time the role of miR-9 to affect the growth of these liver tumor spheres and identified that miR-9 overexpression induced the formation of liver spheres derived from SNU-449 cells, suggesting its potential involvement in early stages during HCC oncogenesis. On the other hand, inhibition of miR-9 by an anti-sense microRNA-9 molecule, suppressed the growth of SNU-449-derived tumor spheres.

Bioinformatics and molecular analyses revealed that miR-9 is involved in HCC pathogenesis through direct regulation of CDH1 and PPARA genes, by binding on their 3′UTR regions. Previous studies have shown that reduced expression of CDH1 correlate with poor outcomes in HCC patients [36]. Consistent with our findings, Tan HX et al. showed that miR-9 was significantly up-regulated in primary HCC tumors with metastases in comparison with those without metastases [37]. In the same study, CDH1 levels were found to be up-regulated after miR-9 inhibition. Other studies have shown that high levels of CDH1 have been correlated with suppression of liver carcinogenesis [38]. In addition, we found that miR-9 overexpression resulted in increased vimentin levels, which is a well-known mesenchymal marker correlated with CDH1 loss of expression in HCC [39]. More importantly, the role of PPARA in HCC pathogenesis has not been previously described. PPARA is a transcription factor that has been implicated in hepatic steatosis [40] and hepatic metabolic homeostasis through regulation of the hepatocyte nuclear factor-4 alpha (HNF4A) gene [41]. Interestingly, we have recently found that HNF4A is a tumor suppressor gene in HCC pathogenesis [7]. Furthermore, it has been described that there is a positive correlation between CDH1 and the PPARA signaling pathways [17, 18]. Our analysis revealed that there is not only a positive correlation between PPARA and CDH1 mRNA levels in HCC, but also that PPARA regulates CDH1 mRNA expression levels in HCC. This observation is very interesting and novel, since miR-9 is using two discrete molecular pathways to suppress CDH1 expression in HCC. First, miR-9 directly suppresses CDH1 mRNA levels through binding on its 3′UTR and in the second indirect mechanism miR-9 suppresses PPARA mRNA levels directly, resulting in decreased CDH1 levels. Overall, these data suggest that microRNAs could use complementary mechanisms to regulate a specific downstream signaling target.

Recent studies have shown that manipulation in the expression levels of microRNAs could have therapeutic potential *in vitro* and *in vivo*. Specifically, administration of miR-26a or miR-124a has resulted in suppression of liver cancer tumor growth in vivo [7, 42]. On the other hand, miR-21 inhibition suppresses HCC growth [43]. Here, we have found that miR-9 inhibition of expression by an antisense-miR-9 suppressed the ability of liver cancer cells to form colonies in soft agar, tumor spheres and decreased their invasiveness, suggesting that targeting miR-9 could be a promising strategy to be further evaluated for the treatment of HCC.

## Conclusions

Integration of high throughput microRNA library screening and microRNA profiling in HCC tissues revealed that miR-9 has both functional and clinical significance in HCC. Furthermore, we found that miR-9 exerts its oncogenic activities through direct regulation of PPARA and CDH1 genes. In addition, we provided evidence that inhibition of miR-9 suppresses HCC cell growth and invasiveness. Taken together, our study identified a novel microRNA signaling pathway, consisting of miR-9, PPARA and CDH1 that is deregulated in HCC patients affecting liver cancer cellular invasiveness.

## Additional file

Additional file 1: Figure S1. Relative (**A**) miR-21 and (**B**) miR-224 expression levels in HCC tumors in different stages (I,II,III,IV) assessed by real-time RT-PCR analysis and normalized to control liver tissues. Data are represented as mean ± SE. ****P* < 0.001, in comparison to control. **Figure S2.** Effects of miR-9 overexpression on HepG2 cancer properties. (**A**) Relative miR-9 expression levels in HepG2 cells after transfection with miR control or miR-9, 48 h post-transfection. (**B**) Cell growth of HepG2 liver cancer cells transfected with miR negative control (miR-Ctrl) or miR-9, 48 h and 72 h post-transfection. (**C**) Invasion of HepG2 after transfection with miR negative control (miR-Ctrl) or miR-9, 48 h post-transfection. (**D**) Soft agar colony assay in HepG2 cells overexpressing miR negative control (miR-Ctrl) or miR-9. All data are represented as mean ± SE. ****P* < 0.001, ***P* < 0.01. **Figure S3.** PPARA relative mRNA levels after CDH1 inhibition in SNU-449 cells. PPARA mRNA levels were measured by qPCR analysis in SNU-449 cells transfected with an siRNA negative control (si-NC) and an siRNA against CDH1 (si-CDH1), 48 h post-transfection. (PPTX 293 kb)
