# Supplementary material for: Functional microRNA high throughput screening reveals miR-9 as a central regulator of liver oncogenesis by affecting the PPARA-CDH1 pathway
Source: BMC Cancer. 2015 Jul 24;15:542. doi: 10.1186/s12885-015-1562-9 (PMC4512159; doi:10.1186/s12885-015-1562-9)
Supplement: Additional file 1: Figure S1. — Relative (A) miR-21 and (B) miR-224 expression levels in HCC tumors in different stages (I,II,III,IV) assessed by real-time RT-PCR analysis and normalized to control liver tissues. Data are represented as mean ± SE. ***P < 0.001, in comparison to control. Figure S2. Effects of miR-9 overexpression on HepG2 cancer properties. (A) Relative miR-9 expression levels in HepG2 cells after transfection with miR control or miR-9, 48 h post-transfection. (B) Cell growth of HepG2 liver cancer cells transfected with miR negative control (miR-Ctrl) or miR-9, 48 h and 72 h post-transfection. (C) Invasion of HepG2 after transfection with miR negative control (miR-Ctrl) or miR-9, 48 h post-transfection. (D) Soft agar colony assay in HepG2 cells overexpressing miR negative control (miR-Ctrl) or miR-9. All data are represented as mean ± SE. ***P < 0.001, **P < 0.01. Figure S3. PPARA relative mRNA levels after CDH1 inhibition in SNU-449 cells. PPARA mRNA levels were measured by qPCR analysis in SNU-449 cells transfected with an siRNA negative control (si-NC) and an siRNA against CDH1 (si-CDH1), 48 h post-transfection. (PPTX 293 kb) [file 12885_2015_1562_MOESM1_ESM.pptx]

## Slide 1
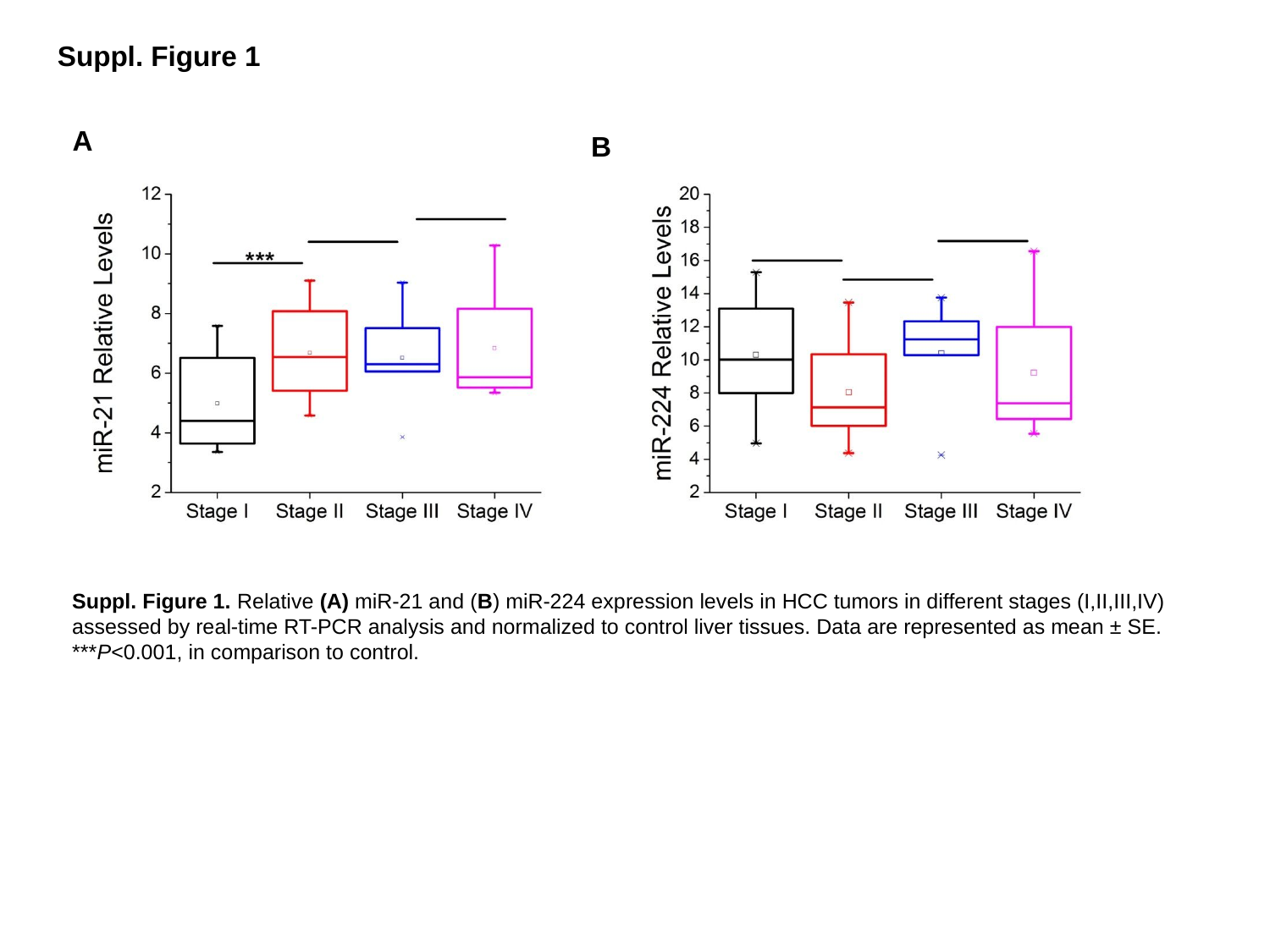

Suppl. Figure 1
A
B
Suppl. Figure 1. Relative (A) miR-21 and (B) miR-224 expression levels in HCC tumors in different stages (I,II,III,IV) assessed by real-time RT-PCR analysis and normalized to control liver tissues. Data are represented as mean ± SE. ***P<0.001, in comparison to control.

## Slide 2
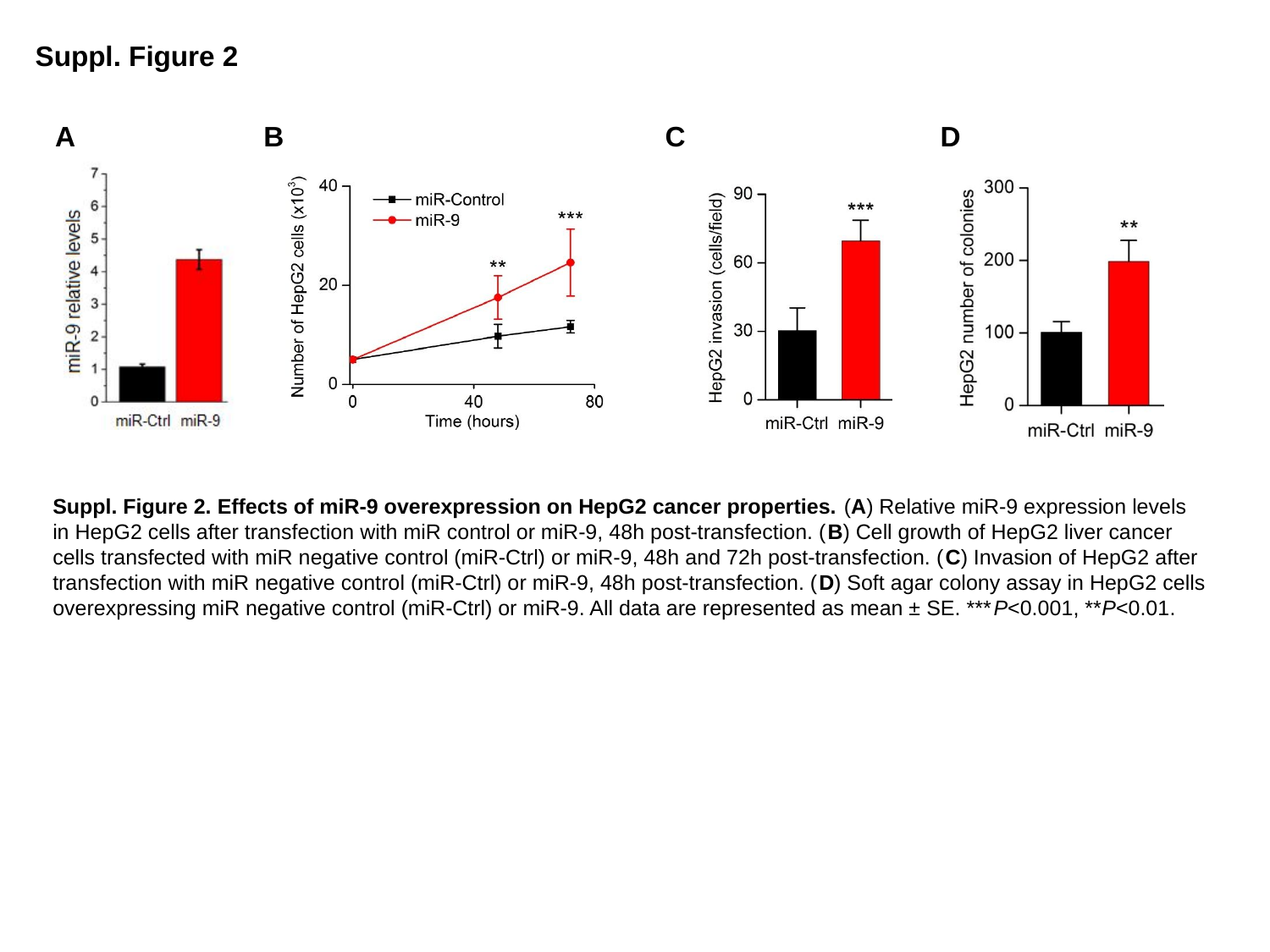

Suppl. Figure 2
A
B
C
D
Suppl. Figure 2. Effects of miR-9 overexpression on HepG2 cancer properties. (A) Relative miR-9 expression levels in HepG2 cells after transfection with miR control or miR-9, 48h post-transfection. (B) Cell growth of HepG2 liver cancer cells transfected with miR negative control (miR-Ctrl) or miR-9, 48h and 72h post-transfection. (C) Invasion of HepG2 after transfection with miR negative control (miR-Ctrl) or miR-9, 48h post-transfection. (D) Soft agar colony assay in HepG2 cells overexpressing miR negative control (miR-Ctrl) or miR-9. All data are represented as mean ± SE. ***P<0.001, **P<0.01.

## Slide 3
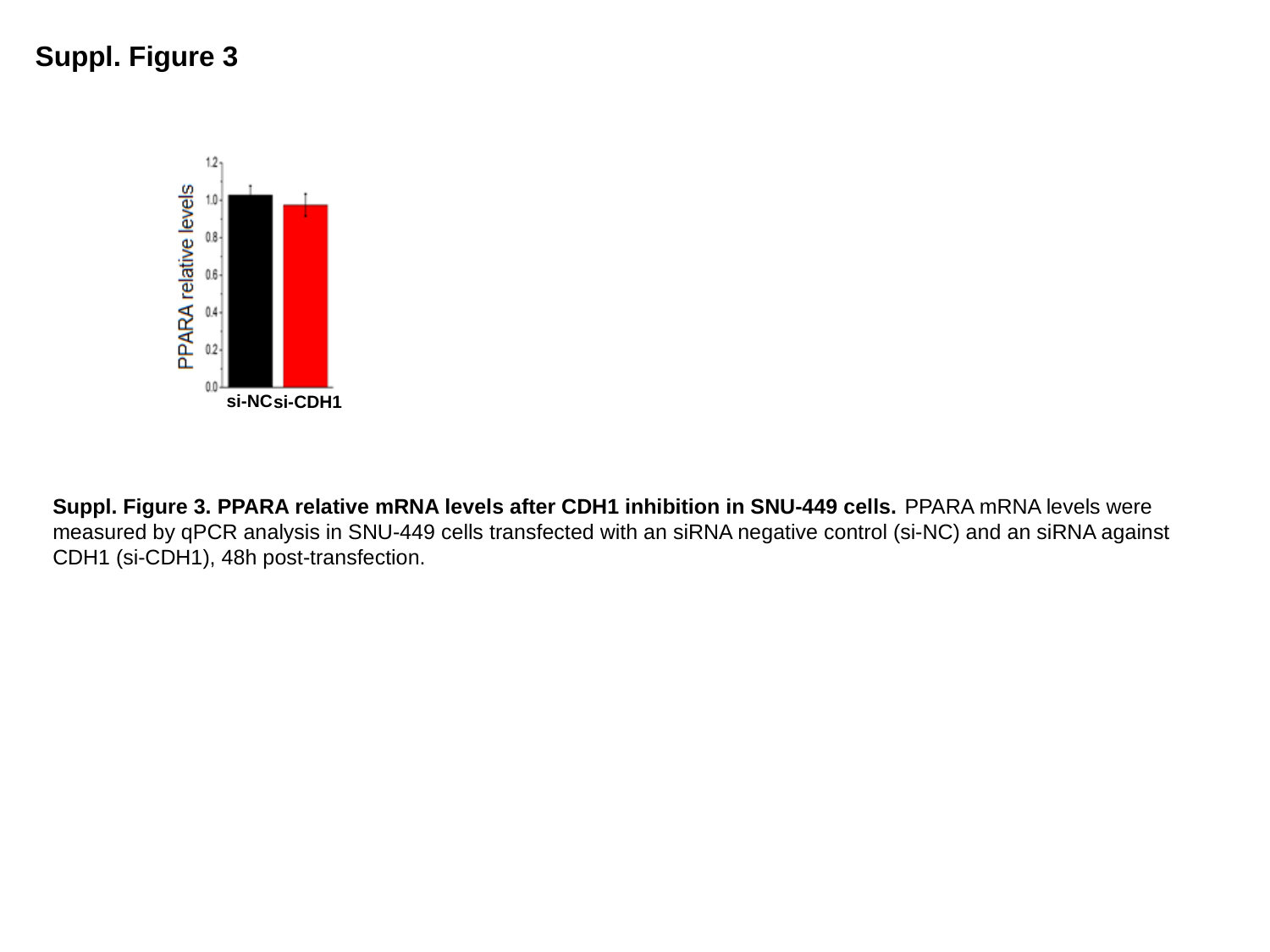

Suppl. Figure 3
si-NC
si-CDH1
Suppl. Figure 3. PPARA relative mRNA levels after CDH1 inhibition in SNU-449 cells. PPARA mRNA levels were measured by qPCR analysis in SNU-449 cells transfected with an siRNA negative control (si-NC) and an siRNA against CDH1 (si-CDH1), 48h post-transfection.
